# Psychometric validation of four-item exercise identity and healthy-eater identity scales and applications in weight loss maintenance

**DOI:** 10.1186/s12966-024-01573-y

**Published:** 2024-02-23

**Authors:** Ann E. Caldwell, Kimberly R. More, Tsz Kiu Chui, R. Drew Sayer

**Affiliations:** 1 Division of Endocrinology, Metabolism, & Diabetes, Anschutz Health and Wellness Center, University of Colorado Anschutz School of Medicine, 12348 E. Montview Blvd, 80045 Aurora, CO USA; 2 Department for Health, University of Bath, Bath, UK; 3 Department of Nutrition Sciences, University of Alabama at Birmingham, Birmingham, AL USA; 4 Department of Family and Community Medicine, University of Alabama at Birmingham, Birmingham, AL USA

**Keywords:** Weight loss maintenance, Health promotion, Exercise, Healthy eating, Identity

## Abstract

**Background:**

Identifying as someone who engages in health promoting behaviors like healthy eating and exercising may be associated with sustained engagement in those behaviors, but reliable and valid instruments are needed to improve the rigor of this research. Two studies were conducted to (1) examine the psychometric properties of a four-item exerciser identity measure (4-EI) and an adapted healthy-eater identity measure (4-HEI) and (2) examine differences in identity strengths across categories of weight loss success.

**Methods:**

Data from 1,709 community dwelling adults in the International Weight Control Registry (IWCR) were used. A random half of the sample was used to assess the proposed unidimensional factor structure of the 4-EI and 4-HEI and examine convergent and discriminant validity using Spearman rank-order correlations. One-way ANOVA was used in the other random half of the sample to compare 4-EI and 4-HEI scores (-3 to + 3) across three self-defined weight loss categories (‘Successful’, ‘Regain’, and ‘Unsuccessful’) and those maintaining ≥ 5% weight loss for > 1 year vs. not.

**Results:**

Results support the unidimensional factor structure with all four items (eigenvalue scores > 2.89) as well as convergent and discriminant validity for both measures. Exercise identity was strongly correlated with self-reported physical activity (*r* (735) = 0.52, *p* <.001) and measures of autonomous motivation. Healthy eating identity was moderately correlated with cognitive restraint in eating (*r* (744) = 0.42, *p* <.001) and other measures predictive of eating behavior. 4-EI and 4-HEI are stronger in Successful (4-EI: *M =* 0.90, *SD =* 1.77; 4-HEI: *M =* 1.56 *SD =* 1.37) vs. Regain (4-EI: *M=-*0.18, *SD =* 1.68; 4-HEI: *M =.*57, *SD =* 1.48) and Unsuccessful (4-EI:*M=*-0.28, *SD =* 1.62; 4-HEI: *M =* 0.51, *SD =* 1.33) and those maintaining ≥ 5% weight loss (4-EI:*M =* 0.47, *SD =* 1.78; 4-HEI: *M =* 1.13, *SD =* 1.49) vs. not (4-EI:*M=*-0.27, *SD =* 1.66; 4-HEI: *M =* 0.53, *SD =* 1.47), *p’s* < 0.001.

**Conclusions:**

The 4-EI and 4-HEI have acceptable psychometric properties and can advance understanding of the role of identity in exercise and dietary behaviors and weight loss maintenance.

**Trial registration:**

The parent observational study, International Weight Control Registry (IWCR), for these sub-studies is registered in ClinicalTrials.gov (NCT04907396).

**Supplementary Information:**

The online version contains supplementary material available at 10.1186/s12966-024-01573-y.

## Introduction

Maintaining a healthy body weight, consuming a healthy diet, and engaging in regular physical activity reduce the occurrence of heart disease, type 2 diabetes mellitus, and several types of cancers [1, 2]. Despite the many well-known benefits of such health-promoting behaviors, sustaining regular engagement in healthy dietary and physical activity behaviors beyond six months, a common threshold for behavior change maintenance, is difficult for most individuals [3–5]. Similarly, behavioral weight loss trials that emphasize changes in dietary and/or physical activity behaviors are characterized by initial and clinically meaningful weight loss at six months (i.e., ≥ 5% mean weight loss) followed by a predictable pattern of weight regain in most participants [5, 6]. At the population level, physical inactivity in US adults is increasing (up to 47.7% in 2020- 7), only 10% of American adults consume the recommended amount of fruits and vegetables [8], and 90% of American adults consume too much sodium [2] despite serious efforts to improve engagement with these health promoting behaviors. The consistency of the observed inability for most people to sustain healthy dietary and physical activity behaviors demonstrates that innovative approaches for sustaining health behavior motivation and change are urgently needed.

There is an increasing recognition in the fields of health psychology and behavioral medicine that self-categorization with a health-related behavior, or identity (e.g., exerciser, healthy-eater, or smoker), can influence the sustainability of engaging in health promoting behaviors and cessation of health-risk behaviors [9–12]. A growing body of research has demonstrated that identifying as an exerciser is associated with physical activity engagement, inclusive of exercise, and identifying as a healthy-eater is associated with healthy eating behaviors. According to social identity theory, this occurs as identities include pre-defined standards of behavior that can reflexively regulate behavior through positive affect when behavior aligns with identity, and negative affect when there is discordance between behavior and identity [13–15]. Most research examining the role of identity on behavior has been conducted in the domain of exercise. A meta-analysis of 32 independent datasets found that exercise identity had a moderate association with self-reported exercise behavior [16], including predicting the frequency of weekly exercise, exercise maintenance (i.e., weeks of previous engagement), as well as intentions to exercise (e.g., 17,18). Identity also emerged as a factor that moderated the intention-behavior gap in a recent systematic review [19]. Although there are limited longitudinal studies on the development of exercise identity, a 16-week exercise intervention study in women demonstrated that participants experienced significant increases in exercise identity during the intervention, and those changes positively predicted exercise maintenance six months later [20].

Healthy-eater identity is less well-studied but has been shown to predict both intentions to eat healthfully (e.g., 21) as well as actual eating behaviors, including lower fat consumption, higher fiber consumption [22], and higher fruit and vegetable consumption [11]. Considering these promising associations between identity and health-promoting behaviors, a call has been made to advance the rigor of this research through instrument development and psychometric evaluation of existing measures [23].

The Exercise Identity Scale is the most widely used validated measure of health-behavior identity [17, 23]. Although it only includes nine items, previous psychometric analysis in college students suggests the items assess two distinct factors: exercise-related identity and beliefs. A shorter, four-item measure that has also been used to measure exercise identity [24] may only have one factor and has been shown to be adaptable to other health-related behaviors as it was initially developed to measure identifying as a green consumer [25] and was subsequently adapted to measure other aspects of consumer identity [26] and smoking identity [27]. Using this four-item measure, those with stronger exercise identities were shown to have higher intentions to exercise and were more successful at following through with their intentions compared to those with weaker exercise identities [24, 28]. These results align with those obtained using the nine-item Exercise Identity Scale. However, the psychometric properties of the four-item measure have not been evaluated.

The purpose of the present research is two-fold. First, Study 1 was designed to assess the proposed unidimensional structural validity as well as the convergent and discriminant validity of the four-item exercise identity scale and an adaptation for measuring healthy-eater identity in a large community sample of adults from across the United States. Second, in Study 2 these measures were used to examine the association between weight loss maintenance and exercise and healthy-eater identity as new models of behavior change, including the Maintain IT Model of Health Behavior Change and Maintenance and the Multi-Process Action Control model emphasize the importance of identity formation for transitioning from healthy behavior adoption to long-term maintenance [9, 10]. Thus theoretically, identity should predict long-term maintenance of both exercise and healthy eating, which are predictive of weight loss maintenance [29–31]. Therefore, it was hypothesized that individuals who have successfully maintained weight loss would have stronger exercise identity and healthy-eater identity. Method for Studies 1 and 2.

**Fig. 1:**
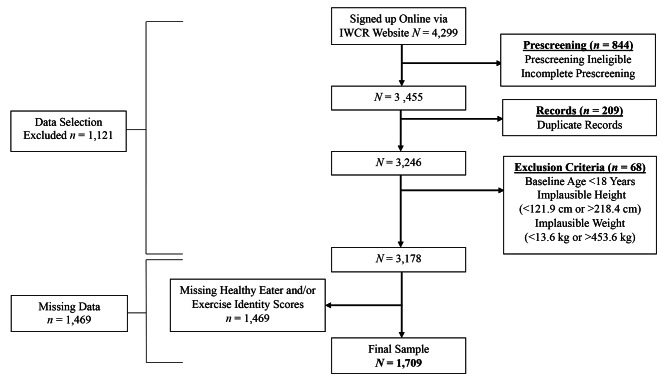
STROBE (Strengthening the Reporting of Observational Studies in Epidemiology) Diagram

This article reports how sample size, all data exclusions, manipulations, and all measures that were included in each study were determined (see Fig. 1; Tables 1, 2 and 3) following the Strengthening the Reporting of Observational Studies in Epidemiology (STROBE Statement) [32]. All analyses were conducted in SPSS v28.0.1. The parent observational study, International Weight Control Registry (IWCR), for these sub-studies is registered in ClinicalTrials.gov (NCT04907396) and the parent study was approved by the institutional review board at Tufts University, but the hypotheses tested in the presented sub-studies were not pre-registered prior to data collection. All participants provided informed consent prior to study participation.

**Table 1:** Study 1: Descriptive Statistics and Correlations for Exercise Identity (*n* = 735)

Variable	N	Mean	SD	Median	IQR	1	2	3	4	5	6	7	8	9	10	11	12	13	14	15	16
1. Exercise identity	735	0.10	1.70	0.00	2.75	-															
2. Healthy eating identity	730	0.70	1.51	0.75	2.50	0.46^**^	-														
3. Total leisure activity (MET ^a^ minute/week)	735	1279.61	2171.79	328.50	1638	0.52^**^	0.25^**^	-													
4. Leisure walking (MET minute/week)	732	453.03	787.29	99.00	594.00	0.36^**^	0.23^**^	0.78^**^	-												
5. Leisure vigorous physical activity (MET minute/week)	735	570.18	1310.91	0.00	360.00	0.47^**^	0.17^**^	0.71^**^	0.30^**^	-											
6. Leisure moderate physical activity (MET minute/week)	731	259.67	651.13	0.00	160.00	0.38^**^	0.19^**^	0.64^**^	0.37^**^	0.50^**^	-										
7. BREQ3^b^ Identified regulation	729	2.59	1.02	2.75	1.75	0.75^**^	0.43^**^	0.50^**^	0.36^**^	0.42^**^	0.35^**^	-									
8. BREQ3 Integrated regulation	727	1.98	1.30	2.00	2.00	0.81^**^	0.47^**^	0.55^**^	0.40^**^	0.45^**^	0.38^**^	0.85^**^	-								
9. BREQ3 Intrinsic regulation	730	2.08	1.22	2.25	2.00	0.66^**^	0.36^**^	0.45^**^	0.31^**^	0.37^**^	0.31^**^	0.74^**^	0.79^**^	-							
10. BREQ3 Introjected regulation	732	2.04	1.17	2.00	1.88	0.35^**^	0.16^**^	0.20^**^	0.11^**^	0.20^**^	0.12^**^	0.50^**^	0.47^**^	0.39^**^	-						
11. BREQ3 Amotivation	731	0.45	0.78	0.00	0.75	-0.39^**^	-0.21^**^	-0.25^**^	− 0.019^**^	-0.18^**^	-0.16^**^	-0.51^**^	-0.46^**^	-0.44^**^	-0.23^**^	-					
12. BREQ3 External regulation	729	0.69	0.88	0.25	1.00	0.02	-0.01	0.06	0.06	0.05	0.05	-0.01	0.03	-0.08^*^	0.24^**^	0.24^**^	-				
13. Age (years)	660	52.09	13.90	53.00	21.50	0.02	-	-	-	-	-	-	-	-	-	-	-	-			
14. Worry about infected with COVID-19	665	2.72	0.97	3.00	1.00	0.05	-	-	-	-	-	-	-	-	-	-	-	-0.02	-		
15. Numbers of people live in household	731	1.93	1.55	2.00	2.00	-0.05	-	-	-	-	-	-	-	-	-	-	-	-0.25^**^	0.03	-	
16. Height (meters)	733	1.66	0.08	1.65	0.13	-0.10^**^	-	-	-	-	-	-	-	-	-	-	-	0.00	0.03	-0.03	-

^a^ **MET = Metabolic Equivalent Task**

^b^ BREQ-3 = Behavior Regulations in Exercise Questionnaire-3, ^*^p <.05. ^**^p <.01

**Table 2:** Study 1: Descriptive Statistics and Correlations for Healthy Eating Identity (*n* = 834)

Variable	N	Mean	SD	Median	IQR	1	2	3	4	5	6	7	8	9
1. Healthy eating identity	834	0.70	1.50	0.75	2.50	-								
2. TFEQ^a^ Cognitive restraint	774	10.86	4.47	11.00	6.00	0.42^**^	-							
3. TFEQ Uncontrolled eating	779	8.79	3.95	13.00	6.00	-0.30^**^	-0.15^**^	-						
4. TFEQ Emotional eating	777	5.77	3.70	2.00	6.00	-0.28^**^	-0.20^**^	0.65^**^	-					
5. Food Craving	800	47.77	16.96	50.00	23.00	-0.27^**^	-0.17^**^	0.77^**^	0.67^**^	-				
6. Age (years)	741	51.90	13.90	53.00	22.00	0.18^**^	-	-	-	-	-			
7. Worry about infected with COVID-19	663	2.73	0.97	3.00	1.00	0.02	-	-	-	-	-0.02	-		
8. Numbers of people live in household	830	1.93	1.55	1.00	2.00	-0.02	-	-	-	-	-0.25^**^	0.03	-	
9. Height (m)	832	1.67	0.09	1.65	0.13	-0.03	-	-	-	-	0.00	0.03	-0.03	-

^a^TFEQ = Three-Factor Eating Questionnaire; ^b^ Food Craving = Food Craving Questionnaire Trait– Reduced, ^*^p <.05. ^**^p <.01

**Table 3:** Study 2: Identity and weight loss by weight loss categories

	Weight Loss Category			
	Self-defined			
	**Successful**	**Regain**		**Unsuccessful**
Exercise identity strength	0.90 (1.77)	− 0.18 (1.68)		− 0.28 (1.62)
Healthy-eater identity strength	1.56 (1.37)	0.57 (1.48)		0.51 (1.33)
Weight loss (%)	-19.09 (12.26)	-6.79 (11.67)		-5.21 (7.95)
Maintaining ≥ 5% WL, n (%)	268 (94%)	243 (51%)		27 (38%)
Not maintaining ≥ 5% WL, n (%)	17 (6%)	229 (49%)		45 (63%)
	**Defined by Reported Weights**			
	**Maintaining ≥ 5% WL**		**Not maintaining ≥ 5% WL**	
Exercise identity strength	0.47 (1.79)	− 0.34 (1.65)		
Healthy-eater identity strength	1.13 (1.48)	0.49 (1.46)		
Weight loss (%)	-16.41 (10.51)	− 0.67 (10.93)		

Abbreviations: Weight loss (WL)

Mean (SD) presented unless otherwise noted

Exercise identity: *n* = 254 self-defined ‘Successful’, *n* = 417 self-defined ‘Regain’, *n* = 65 self-defined ‘Unsuccessful’; *n* = 477 maintaining ≥ 5% WL; *n* = 259 not maintaining ≥ 5% WL

Healthy-eater identity: *n* = 283 self-defined ‘Successful’; *n* = 472 self-defined ‘Regain’, *n* = 72 self-defined ‘Unsuccessful’; *n* = 536 maintaining ≥ 5% WL; *n* = 291 not maintaining ≥ 5% WL

Weight loss: *n* = 284 self-defined ‘Successful’; *n* = 470 self-defined ‘Regain’; *n* = 72 self-defined ‘Unsuccessful’; *n* = 536 maintaining ≥ 5% WL, *n* = 290 not maintaining ≥ 5% WL

## Population

Data for these studies were collected among adults who enrolled in IWCR (described in detail in 33) between December 2020 and October, 2021. The IWCR is a weight control registry that includes individuals from the United States (US), Kuwait, Italy and Greece who have been involved or interested in weight loss. Inclusion criteria for IWCR were at least 18 years of age and have attempted or are planning weight loss. Only participants resided in the US were included in the presented studies. Participants were recruited through email, flyers and social media posts from clinical trials relevant to obesity, recruitment databases, affiliated primary care networks and weight management centers, and community partners. Some participants also entered the IWCR directly from the public IWCR study website (https://internationalweightcontrolregistry.org/). After providing informed consent and enrolling, participants completed a large battery of self-report online questionnaires to measure weight history, dietary and exercise behaviors, psychosocial predictors of both eating and exercise, and weight loss/management, including the measures in the presented studies. Data were collected and managed using REDCap electronic data capture tools hosted at the University of Alabama at Birmingham [34, 35]. For the present studies, participants were excluded if they reported age below 18 years and implausible data on height (< 121.9 cm and > 218.4 cm) and weight (< 13.6 kg and > 453.6 kg) [36, 37]. The full sample included 1,709 participants who completed either the healthy eating identity questionnaire and/or the exercise identity questionnaire. See Fig. 1. for data selection. A stratified random sample was completed by selecting cases at random for inclusion in either study 1 or 2. Approximately half of the sample was used for the psychometric validation study, Study 1 (*n* = 839), and the other half for Study 2 (*n* = 870).

## Study 1 method

### Measures

#### Identity

Exercise identity was assessed using a four-item scale (4-EI; 24) and the four items were adapted to create a healthy eating identity scale (4-HEI; e.g., “I see myself as someone who engages in ‘sufficient exercise’/’healthy eating’” (see Table 4 for all four items). Responses on the 7-point Likert scale range from − 3 (*strongly disagree*) to + 3 (*strongly agree*). Scores from the four items were averaged and scale scores range from − 3 to + 3.

**Table 4:** Study 1: Exploratory Factor Analysis for Exercise Identity and Healthy Eating Identity

	Factor Loading	Extraction Communality
**Exercise Identity Items (** ***n*** ** = 735)**		
‘Engaging in sufficient exercise is something that fits the way I want to live’	0.472	0.223
‘Engaging in sufficient exercise is something that fits who I am’	0.734	0.539
‘I see myself as someone who engages in sufficient exercise’	0.944	0.894
‘I am a typical person who engages in sufficient exercise’	0.934	0.876
**Health Eating Identity Items (*n*** **= 834)**		
‘Engaging in healthy eating is something that fits the way I want to live’	0.546	0.298
‘Engaging in healthy eating is something that fits into who I am’	0.808	0.652
‘I see myself as someone who engages in healthy eating’	0.933	0.871
‘I am a typical person who engages in healthy eating’	0.888	0.789

#### Physical activity

The International Physical Activity Questionnaire (IPAQ) long form was used to measure physical activity as it has demonstrated reliability and validity in 12 countries worldwide [38]. Only physical activity in the leisure domain was used in the present study, including total leisure activity (Metabolic Equivalent Task [MET] minutes/week), leisure walking (MET minutes/week), leisure vigorous physical activity (MET minutes/week), and leisure moderate physical activity (MET minutes/week). The leisure domain was the sole focus as it captures intentional physical activity (i.e., “exercise”), which is conceptually distinct from utilitarian or obligatory physical activity for work or transportation. Calculations of MET min/week for each category were based on the IPAQ scoring protocol [39]. First, any activity that was engaged in for fewer than 10 min was re-coded to 0 min. Second, time spent on leisure walking (minutes/day), leisure vigorous physical activity (minutes/day), and leisure moderate physical activity (minutes/day) were truncated to 180 min/day per activity if participants reported time spent on these activities were over 180 min/day. Third, MET minutes/week were calculated by multiplying MET values to minutes/week of physical activity for each category (leisure walking, leisure moderate physical activity, and vigorous physical activity [39]. MET values were 3.3 for walking, 4.0 for moderate intensity, and 8.0 for vigorous intensity physical activity (IPAQ, 2005).

#### Exercise motivation

The Behavioral Regulations in Exercise-3 (BREQ-3) subscales were used to measure exercise motivation types along the amotivation-autonomous continuum as defined by Self Determination Theory [40–43].

#### Eating behaviors

Self-reported eating behaviors were assessed with the widely used three-factor eating questionnaire [44], which includes three subscales: cognitive restraint, unrestrained eating, and emotional eating, as well as the short version of the Food Craving Questionnaire– Trait [45].

#### Discriminant validity variables

Self-reported height, age, number of household co-residents, and worry about contracting COVID-19 were used as continuous or ordinal variables that were not predicted to be related to either exercise or healthy eating identity. Worry about contracting COVID-19 was assessed with one item, “How worried have you been about becoming infected with COVID-19?” on a scale from 1 (Extremely worried) to 5 (I have wanted to get infected). This question included “Not Worried” as response option #4 to represent a typical range of range of responses to a Likert-style question. The extreme option of “I have wanted to get infected” was included in the questionnaire packet because some individuals early in the pandemic considered using intentional exposure to the SARS-CoV-2 virus to induce natural immunity [46].

#### Socio-demographic characteristics

Socio-demographic characteristics included age in years (calculated as differences between years of birth and year of baseline assessment in 2021), biological sex (male and female), education (12th grade or GED, some college/associate degree, college degree(s), nondoctoral graduate degree, and doctoral degree), body mass index (BMI) (kg/m^2^), race (American Indian/Alaska Native, Asian, Native Hawaiian/other Pacific Islander, Black/African American, White/Caucasian, more than one race, and other), ethnicity (Hispanic or Latino, Hispanic/Latino), household income (less than $25,000, $25,000–$49,999, $50,000–$79,999, $80,000–$130,000, and greater than $130,000), and US census region (Northeast, West, South, and Midwest). Quintiles of household income were created based on the quantile of national household income in 2019 [47]. US census region was grouped based on the zip codes reported by participants. Participants had the option to choose response including “decline to answer”, “prefer not to specify”, and/or “unknown” for questions about biological sex, race, and ethnicity. Missingness of data were minimal (between *n* = 1 to *n* = 13), and in a sample of this size is reasonable to assume was missing at random.

### Statistical analyses

A maximum likelihood exploratory factor analysis was conducted using parallel analysis and an oblique (i.e., correlated) rotation to determine whether the four exercise identity items and four healthy eating identity items should each be combined into one scale. Due to several variables having a skewed distribution, convergent and discriminant validity were assessed with Spearman rank-order correlations between exercise identity and healthy eating identity with factors that were predicted to be associated with each identity, and measures predicted to be less strongly associated, or not associated, with each identity (predictions outlined in Supplemental Table 1).

## Study 1 results

### Participants

Sample characteristics for participants who completed the identity questionnaires are shown in Table 5. For Study 1, participants were on average 52 (*SD* = 14) years of age and had an average Body Mass Index (BMI) of 33.6 kg/m^2^ (*SD* = 8.4). A majority of participants identified as female (87%) and reported race/ethnicities included 74% White, 18% Black, and 7% Hispanic.

**Table 5:** Participant Characteristics

	Study 1 *n* = 839	Study 2 *n* = 870
Age, mean (*SD*)	52 (14)^a^	52 (14)^b^
BMI, mean (*SD*) kg/m^2^	33.6 (8.4)^a^	32.8 (8.3)
Weight Loss Category, *n* (%)		
Successful	226 (27)	285 (33)
Regain	502 (60)	472 (54)
Unsuccessful	74 (9)	72 (8)
First Time Attempting Weight Loss	37 (4)	41 (5)
Sex, *n* (%)		
Female	730 (87)	728 (84)
Race, *n* (%)		
White	621 (74)	642 (74)
Black	154 (18)	149 (17)
Other	63 (8)	78 (9)
Ethnicity, *n* (%)		
Not Hispanic or Latino	773 (93)	904 (93)
Education, *n* (%)		
12th grade or GED	61 (7.3)	46 (5.3)
Some college/Associate degree	194 (23.2)	218 (25.1)
College degree(s)	300 (35.8)	302 (34.8)
Non-doctoral graduate degree	194 (23.2)	210 (24.2)
Doctoral degree	88 (10.5)	91 (10.5)
Household Income, *n* (%)		
Less than $25,000	63 (8)	89 (10)
$25,000-$49,999	160 (19)	154 (18)
$50,000-$79,999	218 (26)	221 (26)
$80,000-$130,000	203 (25)	223 (26)
Greater than $130,000	182 (22)	171 (20)
Region, *n* (%)		
Northeast	141 (17)	169 (20)
Midwest	146 (17)	124 (14)
South	454 (54)	456 (53)
West	94 (11)	115 (13)

^a^For Study 1 age *n* = 743 and BMI *n* = 834 (*n* = 2 were excluded due to implausible values)

^b^For Study 2 age *n* = 792

### Exercise identity

Both the Kaiser-Meyer-Olin Measure of sampling adequacy (0.71), and the Bartlett’s Test of Sphericity (*χ*^2^ = 2,017.57, *p* <.001) suggested that the data were suitable for factor analysis. A parallel analysis [48] revealed that only one factor should be retained. The factor had an eigenvalue score greater than one (i.e., 2.89) and explained 72% of the total variance (Table 4). This factor was composed of all four exercise identity items and factor loadings ranged from 0.47 to 0.94, therefore exceeding the threshold for retention (0.30-0.40) [49], with extracted communalities ranging from 0.22 to 0.89 (see Table 4). The four items had high internal consistency (*α = 0.87).*

Next, convergent validity of the exercise identity measure was assessed through comparing the association between identity and the self-reported physical activity variables, and the autonomous self-determination theory constructs most strongly predictive of exercise: identified, integrated, and intrinsic motivation. As anticipated (Supplemental Tables 1 and Table 1), exercise identity strength was positively associated with total MET-minutes of physical activity (*p* <.001), as well as MET-minutes of vigorous intensity physical activity (*p* <.001), moderate intensity physical activity (*p* <.001), and walking per week (*p* <.001). Additionally, as anticipated, exercise identity was strongly and positively associated with identified (*p* <.001), integrated (*p* <.001), and intrinsic motivation (*p* <.001) for exercise.

In addition to convergent validity, discriminant validity of the exercise identity measure was also assessed (Supplemental Tables 1 and Table 1). It was anticipated that exercise identity would be negatively related to or not related to less internalized forms of exercise-regulation. In line with expectations, exercise identity was negatively related to amotivation towards exercise (*p* <.001) and not related to extrinsic regulation for exercise (*p* =.54). However, counter to expectations, exercise identity was positively associated with introjected motivation towards exercising (*p* <.001). It should be noted that this relationship was weaker than the relationship between exercise identity and the more autonomous forms of motivation. In line with predictions, exercise identity was not related to participants worrying about getting COVID-19 (*p* =.21), the number of co-residents in household (*p* =.19), or age (*p* =.55) but was related to height (*p* =.01).

### Healthy-eater identity

A maximum likelihood exploratory factor analysis was conducted using an oblique (i.e., correlated) rotation to determine whether the four healthy eating identity items should be combined into one scale. Both the Kaiser-Meyer-Olin Measure of sampling adequacy (0.77), and the Bartlett’s Test of Sphericity (*χ*^2^ = 2,105.98, *p* <.001) suggest that the data are suitable for factor analysis. A parallel analysis [48] revealed that only one factor should be retained. The factor had an eigenvalue score greater than one (i.e., 2.96) and explained 73% of the total variance (Table 4). This factor was composed of all four healthy eating identity items and factor loadings ranged from 0.55 to 0.93, exceeding the threshold for retention (0.30-0.40) [49], with extracted communalities ranging from 0.30 to 0.87 (see Table 4). The four items had high internal consistency (*α = 0.88).*

Next, the convergent validity of the healthy-eater identity measure was examined by comparing the association between identity and cognitive restraint, uncontrolled eating, emotional eating, and food cravings (Supplemental Tables 1 and Table 2). As anticipated, healthy-eater identity was positively associated with cognitive restraint (*p* <.001) and negatively associated with uncontrolled eating (*p* <.001), emotional eating (*p* <.001), and food cravings (*p* <.001).

In addition to convergent validity, the discriminant validity of the healthy-eater identity measure was also assessed (Supplemental Tables 1 and Table 2). As predicted, healthy-eater identity was weakly or not associated with participants worrying about getting COVID-19 (*p* =.59), the number of household co-residents (*p* =.49), and height (*p* =.45), though it was significantly associated with age (*p* <.001).

## Study 1 discussion

Findings from this large sample of community dwelling adults from across the United States support the internal reliability and structural, convergent, and discriminant validity of four-item measures of exercise identity and healthy-eater identity. Both scales have a unidimensional factor structure containing all four respective items. Nearly all predictions for convergent and discriminant validity were supported, with one exception. Exercise identity correlated positively with introjected motivation from the BREQ-3 questionnaire, which is designed to measure exercising to avoid guilt. Although introjected motivation is a less autonomous form of motivation within self-determination theory, it aligns with the social identity theory perspective that identity helps behavior to be self-reinforcing as a result of the negative emotions that surface when you fail to act in line with the standards of a given identity [13–15].

It should be noted that one item on each scale had a lower factor loading compared to the other three items, suggesting there is still some room for improvement in the measurement of identity (exercise identity: ‘Engaging in sufficient exercise is something that fits the way I want to live’; Healthy-eater Identity: ‘Engaging in healthy eating is something that fits the way I want to live’). However, both items loaded moderately onto their respective factor (i.e., > 0.30), which indicates that there was a moderate correlation between the individual items and the underlying factors [50].

The four-item measure of exercise identity was related to the self-determination theory construct of integrated motivation (*r* =.81; i.e., internalizing motivation to exercise within one’s self-concept), a construct that was relatively recently added to the autonomous motivation continuum in the third iteration of the BREQ. A standard cut-off for assessing severe multicollinearity between variables is *r* =.80 [51]. Further work is warranted to determine the extent to which exercise identity and integrated motivation are indeed tapping into different constructs or if this is a case of the jangle fallacy (i.e., two or more constructs having different names when in fact they are measuring the same construct;,52). Indeed, other research has also reported a severe multicollinearity issue between these constructs using the Exercise Identity Scale (e.g., *r* =.80) [53]. To determine whether exercise-related identity and integrated motivation are separable constructs, more studies are needed to examine the relationship between these variables and whether they are equally predictive of behavior [52]. In this cross-sectional study, the relationships are similar in terms of the outcome of exercise behavior, but integrated motivation had a stronger association with identified motivation than the association between exercise identity and identified motivation (*r* =.85 vs. *r* =.76, respectively). More studies are needed to determine the extent to which exercise identity is a separable predictor of behavior from the constructs on the self-determination theory continuum of autonomous motivation.

## Study 2 method

### Measures

#### Identity

Exercise identity (4-EI) and healthy-eater identity (4-HEI) were assessed in the other random half of the large sample of US adults using the same measures presented for Study 1 and compared across two types of weight loss categories.

#### Weight loss categories

IWCR members self-defined belonging to one of four weight loss categories: [1] lost weight and maintained the weight loss for at least a year (‘Successful’), [2] lost weight in the past but regained most or all back, ‘Regain’, and [3] tried losing weight but not been able to (‘Unsuccessful’), and [4] Interested in losing weight for the first time (‘Interested’). ‘Interested’ participants (*n =* 41) were excluded from this study. Participants also reported their highest adult weight, date they were at highest weight, and current weight. Weight loss category was dichotomously defined as those who were maintaining a clinically meaningful weight loss (≥ 5%) for > 1 year, and those who were not.

### Statistical analyses

Univariate ANOVAs were used to compare mean scores for both identities across self-defined weight loss groups with planned contrasts between the category of ‘Successful’ and both the categories of ‘Regain’ and ‘Unsuccessful’. A separate model compared identity scores between those who were maintaining ≥ 5% weight loss from their highest adult weight to those who were not. ANCOVAs were used to examine self-reported biological sex, age, race, and ethnicity as potential covariates in all models. Assumptions underlying ANOVA (normal distribution, equal variances, and independent data) were met for healthy-eater identity in both group comparisons and for exercise identity in the self-reported group comparison (Skewness <|2| and Kurtosis <|7|, Levene’s test for equal variances (*p* ≥.05); but equal variances was not met in the 5% weight loss group comparison, therefore a Welch t-test was conducted.

## Study 2 results

### Participants

Participants were 52 (*SD* = 14) years of age on average, had an average BMI of 32.8 kg/m^2^ (*SD* = 8.3). The majority identified as female (84%), and self-reported race/ethnicities included 74% White, 17% Black, and 7% Hispanic (Table 5.). Table 3 has the weight management distribution for Study 2, with 33% self-defining as ‘Successful’, 54% as ‘Regainers’, and 8% as ‘Unsuccessful’. The majority (65%) were maintaining a clinically significant weight loss of at least 5% from their highest adult weight for > 1 year.

### Exercise identity

The 4-EI scale was completed by 736 individuals and the four items had high internal consistency (*α = 0.89).* See Table 3 for identity scores across groups.

There were significant differences across self-defined weight loss categories in exercise identity strength (*F*(2,733) = 34.14, *p* <.001) with a medium effect size, η^2^ = 0.09. This overall difference was followed-up with planned contrasts demonstrating that those in the ‘Successful’ category reported maintaining weight loss for more than one year had stronger exercise identity (*M =* 0.90) compared to the ‘Regain’ category (*M =* -0.18), 95% CI for difference (-1.35, − 0.81), *p* <.001, and ‘Unsuccessful’ (*M =* -0.28), 95% CI for difference (-1.65, − 0.72), *p* <.001. Those who were maintaining a ≥ 5% weight loss from their highest weight also had a stronger exercise identity (*M =* 0.47), compared to those not (*M = −* 0.34), *Welch’s t*(566.07) = 37.58, *p* <.001, 95% CI of difference (0.54, 1.07), and a small effect size, η^2^ = 0.05. No covariates were significantly associated with exercise identity scores.

### Healthy-eater identity

The 4-HEI scale was completed by 827 individuals and the four items had high internal consistency (*α = 0.89).* There were significant differences across self-defined weight loss groups in healthy-eater identity strength (*F*(2,824) = 44.89, *p* <.001) with a medium effect size, η^2^ = 0.10. Those in the ‘Successful’ category who had maintained weight loss for more than one year had stronger healthy-eater identity scores (*M =* 1.56) compared to the ‘Regain’ category (*M =* 0.57), 95% CI for difference (-1.20, − 0.77), *p* <.001, and the ‘Unsuccessful’ category (*M =* 0.51), 95% CI for difference (-1.42, − 0.68), *p* <.001. Similarly, those who were maintaining ≥ 5% weight loss had stronger healthy-eater identities (*M* = 1.13) compared to those who were not (*M* = 0.49), *F*(1, 825) = 35.88, *p* <.001, 95% CI of difference (0.43, 0.85), and a small effect size η^2^ = 0.04.

Age and biological sex were significantly associated with healthy-eater identity scores when included as covariates in each model, but race or ethnicity were not. Age was positively associated with healthy-eater identity, while women reported higher healthy-eater identities than men. However, including sex and age in the models did not alter the results for the effects of self-defined or objective weight loss category, therefore ANOVA results are reported for clarity.

## Study 2 discussion

Findings from a large sample of community dwelling adults residing in the United States supported the hypothesis that participants who self-identified as successful at maintaining weight-loss for at least one year had stronger exercise and healthy-eater identities in comparison with individuals who self-identified as those who had lost weight but regained it, or who were unsuccessful at losing weight. Similarly, those who were maintaining a ≥ 5% weight loss from their highest adult weight for longer than 1 year had stronger exercise and healthy-eater identities. Interestingly, half of those who identified as being in the ‘Regain’ category and over one third who identified as ‘Unsuccessful’ at losing weight were maintaining a clinically meaningful weight loss according to their self-reported highest weight and current weight. These hypotheses were supported even after controlling for the individual differences of age, biological sex, race, and ethnicity, indicating that these differences in health behavior identities relation to weight-loss status are not confounded with demographic differences between groups.

Identity is increasingly being recognized for influencing dietary and exercise behaviors, but to date is relatively understudied in behavioral weight loss trials. Results from this study are in line with the Maintain IT Model [9] and suggest behavior-based identities (i.e., exercise identity and healthy-eater identity) should be further investigated in prospective studies and randomized weight loss and maintenance trials.

## General discussion

Findings from these two studies imply important conclusions. First, the findings from Study 1 support the use of the four-item measures of both exercise and healthy-eater identities to help advance the growing literature examining the role of identity in health behavior change and maintenance. Second, the findings from Study 2 support the importance of identity in the process of sustaining dietary and physical activity behavior changes that are predictive of long-term weight loss maintenance. Specifically, stronger healthy-eater identity and exercise identity scores were observed in those who identified as successfully maintaining weight loss compared to those who identified as having regained most of their lost weight and those who identified as never successfully losing weight and were stronger in those who were maintaining ≥ 5% weight loss for > 1 year compared to and those not maintaining ≥ 5% weight loss.

Studies 1 and 2 have limitations that are worth noting. Key among them is that behavior was measured via self-report, and eating behaviors were assessed using the three-factor eating questionnaire and cravings questionnaire rather than detailed reports of dietary intake by 24-hour recalls or food records. However, self-reported measures of physical activity/exercise [54] and dietary intake [55] are notoriously poor, and this limitation extends to nearly all studies in this area of research to date. Thus, more studies are needed that include objective measures of physical activity and eating behavior. Another general limitation of the research is that the cross-sectional observational nature of the study limits causal inferences that can be drawn from these results. More robust longitudinal and randomized experiments will be necessary to establish causality. The IWCR includes a large battery of questionnaires, and those people who completed the full battery may be more conscientious, which could limit the generalizability of the results. However, this limitation is balanced with the strength of having a sample that is larger and more representative of the US population than the convenience samples of college students used in the psychometric validation study of the nine-item exercise identity scale [23].

## Conclusions

Preliminary data supports the notion that identity is a modifiable target for behavior change interventions that can support more long-term behavior changes in both quantitative and qualitative studies [10, 19, 56, 57]. This article contributes to efforts to increase the rigor of the quantitative measurement of both exercise and healthy-eater identity using a four-item measure that has reasonable internal reliability, unidimensional factor structure, convergent, discriminant (Study 1), and was significantly higher among those who were successful at maintaining weight loss compared to those who reported not maintaining weight loss or not losing weight (Study 2).

### Electronic supplementary material

Below is the link to the electronic supplementary material.

Supplementary Material 1

## Data Availability

The datasets analyzed during the current study available from the corresponding author on reasonable request.

## Abbreviations

4-EI: Four-item exercise identity scale
4-HEI: Four-item healthy-eater identity scale
IWCR: International Weight Control Registry

## References

[1] Kyu HH, Bachman VF, Alexander LT, Mumford JE, Afshin A, Estep K et al. Physical activity and risk of breast cancer, colon cancer, diabetes, ischemic heart disease, and ischemic stroke events: systematic review and dose-response meta-analysis for the global burden of Disease Study 2013. 354, BMJ (Online). 2016. 10.1136/bmj.i3857 PMC4979358 27510511

[2] CDC. Poor Nutrition [Internet]. Centers for Disease Control. 2023 [cited 2023 Feb 27]. Available from: https://www.cdc.gov/chronicdisease/resources/publications/factsheets/nutrition.htm.

[3] Prochaska JO, Velicer WF. The transtheoretical model of health behavior change. Am J Heal Promot. 1997;12(1). 10.4278/0890-1171-12.1.38 10170434

[4] Wood W, Neal DT. Healthy through habit: interventions for initiating & maintaining health behavior change. Behav Sci Policy. 2016;2(1).

[5] MacLean PS, Wing RR, Davidson T, Epstein L, Goodpaster B, Hall KD et al. NIH working group report: Innovative research to improve maintenance of weight loss. Obesity [Internet]. 2015;23(1):7–15. 10.1002/oby.20967. 10.1002/oby.20967 PMC5841916 25469998

[6] Stubbs RJ, Lavin JH. The challenges of implementing behaviour changes that lead to sustained weight management. 38, Nutr Bull. 2013.

[7] CDC. CDC Maps America’s high levels of inactivity. Centers for Disease Control; 2020.

[8] Lee-Kwan SH, Moore LV, Blanck HM, Harris DM, Galuska D. Disparities in State-Specific Adult Fruit and Vegetable Consumption — United States, 2015. MMWR Morb Mortal Wkly Rep. 2017;66(45). 10.15585/mmwr.mm6645a1 PMC5726245 29145355

[9] Caldwell AE, Masters KS, Peters JC, Bryan AD, Grigsby J, Hooker SA (2018). Harnessing centred identity transformation to reduce executive function burden for maintenance of health behaviour change: the maintain IT model. Health Psychol Rev.

[10] Rhodes RE. Multi-process Action Control in Physical Activity: a primer. 12, Front Psychol. 2021. 10.3389/fpsyg.2021.797484 PMC8714894 34975698

[11] Strachan SM, Brawley LR (2009). Healthy-eater identity and self-efficacy predict healthy eating behavior: a prospective view. J Health Psychol.

[12] Priebe CS, Beauchamp M, Wunderlich K, Faulkner G. I’m a runner not a smoker: changes in identity as predictors of smoking cessation and physical activity. Psychol Sport Exerc. 2020;49.

[13] Burke PJ (2006). Identity change. Soc Psychol Q.

[14] Stryker S, Burke PJ (2000). The past, present, and future of an identity theory. Soc Psychol Q.

[15] Stets JE, Burke PJ (2000). Identity theory and social identity theory. Social Psychol Q.

[16] Rhodes RE, Kaushal N, Quinlan A (2016). Is physical activity a part of who I am? A review and meta-analysis of identity, schema and physical activity. Health Psychol Rev.

[17] Anderson DF, Cychosz CM. Development of an exercise identity scale. Percept Mot Skills. 1994;78(3). 10.1177/003151259407800313 8084685

[18] Carraro N, Gaudreau P. The role of implementation planning in increasing physical activity identification. Am J Health Behav. 2010;34(3). 10.5993/ajhb.34.3.5 20001187

[19] Rhodes RE, Cox A, Sayar R. What predicts the physical activity intention-behavior gap a systematic review. Volume 56. Annals of Behavioral Medicine; 2022. 10.1093/abm/kaab044 34231844

[20] Gillman AS, Stevens CJ, Bryan AD. Women’s exercise identity increases after a 16-week exercise RCT and is linked to behavior maintenance at follow-up. Psychol Sport Exerc. 2021;54. 10.1016/j.psychsport.2021.101888 PMC7901813 33633498

[21] Sparks P, Guthrie CA (1998). Self-identity and the theory of planned behavior: a useful addition or an unhelpful artifice?. J Appl Soc Psychol.

[22] Kendzierski D, Costello MC. Healthy eating self-schema and nutrition behavior. 34, J Appl Soc Psychol. 2004.

[23] Wilson PM, Muon S. Psychometric properties of the exercise identity scale in a university sample. Int J Sport Exerc Psychol. 2008;6(2).

[24] de Bruijn GJ. Exercise promotion: An integration of exercise self-identity, beliefs, intention, and behaviour. Eur J Sport Sci [Internet]. 2012;12(4):354–66. Available from: http://www.tandfonline.com/doi/abs/10.1080/17461391.2011.568631.

[25] Sparks P, Shepherd R. Self-identity and the theory of Planned Behavior: assesing the role of identification with Green Consumerism. Soc Psychol Q. 1992;55(4).

[26] Smith JR, Terry DJ, Manstead ASR, Louis WR, Kotterman D, Wolfs J. Interaction effects in the theory of planned behavior: the interplay of self-identity and past behavior. J Appl Soc Psychol. 2007;37(11).

[27] n den Putte B, Yzer M, Willemsen MC, de Bruijn GJ (2009). The effects of Smoking Self-Identity and Quitting Self-Identity on attempts to quit smoking. Heal Psychol.

[28] Verkooijen KT, De Bruijn GJ. Exercise self-identity: interactions with social comparison and exercise behaviour. Psychol Heal Med. 2013;18(4). 10.1080/13548506.2012.750727 23323686

[29] Hill JO, Wyatt H, Phelan S, Wing R. The National Weight Control Registry: Is it useful in helping deal with our obesity epidemic? J Nutr Educ Behav [Internet]. 2005;37(4):206–10. Available from: http://www.ncbi.nlm.nih.gov/pubmed/16029692. 10.1016/s1499-4046(06)60248-0 16029692

[30] Ogden LG, Stroebele N, Wyatt HR, Catenacci VA, Peters JC, Stuht J et al. Cluster analysis of the national weight control registry to identify distinct subgroups maintaining successful weight loss. Obesity [Internet]. 2012;20(10):2039–47. Available from: http://www.ncbi.nlm.nih.gov/pubmed/22469954. 10.1038/oby.2012.79 PMC4562400 22469954

[31] Thomas JG, Bond DS, Hill JO, Wing RR (2011). The national weight control registry: a study of successful losers. ACSM’s Heal Fit J.

[32] von Elm E, Altman DG, Egger M, Pocock SJ, Gøtzsche PC, Vandenbroucke JP. The strengthening the reporting of observational studies in epidemiology (STROBE) statement: guidelines for reporting observational studies. Int J Surg. 2014;12(12).

[33] Roberts SB, Das SK, Sayer RD, Caldwell AE, Wyatt HR, Mehta TS et al. Technical report: an online international weight control registry to inform precision approaches to healthy weight management. Int J Obes. 2022;46(9):1728–33. 10.1038/s41366-022-01158-4 PMC9201790 35710944

[34] Harris PA, Taylor R, Thielke R, Payne J, Gonzalez N, Conde JG. A metadata-driven methodology and workflow process for providing translational research informatics support. J Biomed Inf J Biomed Inf. 2009;42(2). 10.1016/j.jbi.2008.08.010 PMC2700030 18929686

[35] Harris PA, Taylor R, Minor BL, Elliott V, Fernandez M, O’Neal L (2019). The REDCap consortium: building an international community of software platform partners. J Biomed Inf.

[36] Koebnick C, Smith N, Huang K, Martinez MP, Clancy HA, Kushi LH. The prevalence of obesity and obesity-related health conditions in a large, multiethnic cohort of young adults in California. Ann Epidemiol. 2012;22(9). 10.1016/j.annepidem.2012.05.006 PMC3418481 22766471

[37] Pyrkov TV, Avchaciov K, Tarkhov AE, Menshikov LI, Gudkov AV, Fedichev PO. Longitudinal analysis of blood markers reveals progressive loss of resilience and predicts human lifespan limit. Nat Commun. 2021;12(1). 10.1038/s41467-021-23014-1 PMC8149842 34035236

[38] Craig CL, Marshall AL, Sjöström M, Bauman AE, Booth ML, Ainsworth BE (2003). International physical activity questionnaire: 12-Country reliability and validity. Med Sci Sports Exerc.

[39] Ipaq. Guidelines for Data Processing and Analysis of the International Physical Activity Questionnaire (IPAQ)– Short and Long Forms. Ipaq. 2005;(November).

[40] Markland D, Tobin V. A modification to the behavioural regulation in exercise questionnaire to include an assessment of amotivation. J Sport Exerc Psychol. 2004.

[41] Mullan E, Markland D, Ingledew DK (1997). A graded conceptualisation of self-determination in the regulation of exercise behaviour: development of a measure using confirmatory factor analytic procedures. Pers Individ Dif.

[42] Wilson PM, Rodgers WM, Loitz CC, Scime G. It’s who I am… really!’ The importance of Integrated Regulation in Exercise Contexts1. J Appl Biobehav Res. 2007.

[43] Ryan RM, Deci EL (2000). Intrinsic and extrinsic motivations: classic definitions and new directions. Contemp Educ Psychol.

[44] Stunkard AJ, Messick S (1985). The three-factor eating questionnaire to measure dietary restraint, disinhibition and hunger. J Psychosom Res.

[45] Meule A, Hermann T, Kübler A. A short version of the food cravings questionnaire-trait: the FCQ-T-reduced. Front Psychol. 2014;5. 10.3389/fpsyg.2014.00190 PMC3940888 24624116

[46] Streiffer R, Killoren D, Chappell RY. The Ethics of Deliberate exposure to SARS-CoV-2 to induce immunity. J Appl Philos. 2021;38(3). 10.1111/japp.12492 PMC8014061 33821073

[47] Semega J, Kollar M, Ashton V, Bane M, Gajewski SS, Chen A et al. Income and poverty in the United States: 2019. 2020.

[48] Zwick WR, Velicer WF. Comparison of five rules for determining the number of components to Retain. Psychol Bull. 1986;99(3). 10.1207/s15327906mbr1702_5 26810950

[49] Floyd FJ, Widaman KF. Factor analysis in the development and refinement of clinical Assessment instruments. Psychol Assess. 1995;7(3).

[50] Tavakol M, Wetzel A. Factor Analysis: a means for theory and instrument development in support of construct validity. Int J Med Educ [Internet]. 2020 [cited 2023 Mar 7];11:245–7. Available from: http://creativecommons.org/licenses/by/3.0. 10.5116/ijme.5f96.0f4a PMC7883798 33170146

[51] Wu S. Multicollinearity in Regression. Why it is a problem? [Internet]. 2020 [cited 2023 Mar 7]. Available from: https://towardsdatascience.com/multi-collinearity-in-regression-fe7a2c1467ea.

[52] Gonzalez O, MacKinnon DP, Muniz FB. Extrinsic convergent validity evidence to prevent Jingle and Jangle Fallacies. Multivar Behav Res. 2021;56(1). 10.1080/00273171.2019.1707061 PMC7369230 31958017

[53] Strachan SM, Fortier MS, Perras MGM, Lugg C. Understanding variations in exercise-identity strength through identity theory and self-determination theory. Int J Sport Exerc Psychol. 2013;11(3).

[54] Luis G, Ferrari Id M, Kovalskys I, Fisberg M, Gó Mez G, Rigotti A et al. Comparison of self-report versus accelerometer– measured physical activity and sedentary behaviors and their association with body composition in Latin American countries. 2020 [cited 2023 Mar 7]; 10.1371/journal.pone.0232420. 10.1371/journal.pone.0232420 PMC7188285 32343753

[55] Dhurandhar NV, Schoeller D, Brown AW, Heymsfield SB, Thomas D, Sørensen TIA et al. Energy balance measurement: When something is not better than nothing. Int J Obes [Internet]. 2015;39(7):1109–13. Available from: http://www.nature.com/articles/ijo2014199. 10.1038/ijo.2014.199 PMC4430460 25394308

[56] Kwasnicka D, Dombrowski SU, White M, Sniehotta F (2016). Theoretical explanations for maintenance of behaviour change: a systematic review of behaviour theories. Health Psychol Rev.

[57] Gillman AS, Bryan AD (2017). Exercise identity and behavior maintenance after a supervised exercise intervention. Ann Behav Med.

