# Supplementary material for: Psychometric validation of four-item exercise identity and healthy-eater identity scales and applications in weight loss maintenance
Source: Int J Behav Nutr Phys Act. 2024 Feb 23;21:21. doi: 10.1186/s12966-024-01573-y (PMC10885534; doi:10.1186/s12966-024-01573-y)
Supplement: Supplementary file 1 — Supplementary Material 1 [file 12966_2024_1573_MOESM1_ESM.docx]

**Supplemental Table 1.**

*Convergent and Discriminant Variable Hypotheses*

|  | | Construct | Predicted Relationship/Direction | | | | |
| --- | --- | --- | --- | --- | --- | --- | --- |
| Exercise Identity - Convergent Validity | | | | |  | | |
|  | | MET-minutes/week of total leisure time physical activity | | | + | | |
|  |  | MET-minutes/week of leisure time vigorous physical activity | | | + | | |
|  |  | MET-minutes/week of leisure time moderate physical activity | | | + | | |
|  |  | MET-minutes of leisure time walking | | | + | | |
|  |  | Identified exercise motivation | | | + | | |
|  |  | Integrated exercise motivation | | | + | | |
|  |  | Intrinsic exercise motivation | | | + | | |
| Exercise Identity - Discriminant Validity | | | | |  | | |
|  | Amotivation | | | | - or n.s. | | |
|  | Extrinsic exercise motivation | | | | - or n.s. | | |
|  | Introjected motivation | | | | - or n.s. | | |
|  | Number of co-residents in household | | | | n.s. | | |
|  | Worry about contracting COVID-19 virus | | | | n.s. | | |
|  | Height | | | | n.s. | | |
|  | Age | | | | n.s. | | |
| Health Eating Identity - Convergent Validity | | | | |  | | |
|  | Cognitive restraint | | | | + | | |
|  | Uncontrolled eating | | | | - | | |
|  | Emotional eating | | | | - | | |
|  | Trait food cravings | | | | - | | |
| Healthy Eating Identity - Discriminant Validity | | | | | |  | |
|  | Number of co-residents in household | | | | | n.s. | |
|  | Worry about contracting COVID-19 virus | | | | | n.s. | |
|  | Height | | | | | n.s. | |
|  | Age | | | | | n.s. | |

*Note.* n.s. = non-significant.

**Supplemental File 1.**

*Four-item scales measuring exercise identity and healthy eating identity.*

**Four-item exercise identity (4-EI)**

Please rate the degree to which you agree with each statement.

-3 -2 -1 0 1 2 3

Totally Totally

Disagree agree

________ Engaging in sufficient exercise is something that fits the way I want to live.

_________ Engaging in sufficient exercise is something that fits into who I am.

_________ I see myself as someone who engages in sufficient exercise

_________ I am a typical person who engages in sufficient exercise

**Four-item health eating identity (4-HEI)**

Please rate the degree to which you agree with each statement.

-3 -2 -1 0 1 2 3

Totally Totally

Disagree agree

________ Engaging in healthy eating is something that fits the way I want to live.

_________ Engaging in healthy eating is something that fits into who I am.

_________ I see myself as someone who engages in healthy eating.

_________ I am a typical person who engages in healthy eating.
